# Supplementary material for: Near-zero metamaterial inspired UHF antenna for nanosatellite communication system
Source: Sci Rep. 2019 Mar 5;9:3441. doi: 10.1038/s41598-019-40207-3 (PMC6401189; doi:10.1038/s41598-019-40207-3)
Supplement: Supplementary file 1 — Article file in pdf format [file 41598_2019_40207_MOESM1_ESM.pdf]

Near-zero metamaterial inspired UHF antenna for nanosatellite communication system

Touhidul Alam[1*], Mohammad Tariqul Islam[1,2*], Mengu Cho[2,3]
[1]Centre of Advanced Electronic & Communication Engineering,
Faculty of Engineering and Built Environment, Universiti Kebangsaan Malaysia,
Bangi, Selangor D.E., 43600 Malaysia
[2]Laboratory of Spacecraft Environment Interaction Engineering (LaSEINE),
Kyushu Institute of Technology (Kyutech), Kitakyushu-shi, Fukuoka, Japan
[3]School of Electrical Electronic Engineering, Nanyang Technological University,
Nanyang Drive, Singapore

Email: touhid13@siswa.ukm.edu.my (TA), tariqul@ukm.edu.my (MTI)

Abstract— **Epsilon-and-mu-near-zero (EMNZ) metamaterial structure inspired UHF antenna for nanosatellite has been proposed in this paper. The antenna consists of 3×2-unit cell array on the ground plane and a meander line radiating patch. Coaxial probe feeding technique has been obtained to excite the antenna. The meander line enables the antenna to resonate at lower UHF band and the metamaterial array is used to make the resonant frequency stable by reducing the coupling effect with metallic nanosatellite structure. The metamaterial structure exhibits EMNZ characteristics from 385 MHz to 488.5 MHz, which facilitates stable resonant frequency and higher antenna efficiency when embedded with nanosatellite structure. The proposed EMNZ inspired antenna has achieved measured impedance bandwidth (S_{11}< -10dB) of 14.92 MHz (391 MHz – 405.92 MHz). The perceptible novelty of this paper is the development of EMNZ metamaterial that significantly improves the UHF antenna's operating frequency stability as well as efficiency for low earth orbit nanosatellite communications.**

Index Terms—**CubeSat, double near zero (DNZ), epsilon-and-mu-near-zero (EMNZ), metamaterial, nanosatellite, UHF**

I. INTRODUCTION

Over the last decade, nanosatellite missions have increased vividly for low earth orbit space missions. This concept has been very enthusiastic to the scientific, private, and government missions due to miniature electronics size with low-cost and low power consumption [1]. Nanosatellite space missions are being fruitful in coastal and inland critical observation of natural disaster, monitoring agri-environmental and agriculture conditions, and space atmosphere observation. Every nanosatellite has some common functions for satellite operations like power system, uplink-downlink communications, and altitude control. The antenna is the key element of the uplink-downlink communications between satellite and earth. The inherent relation between lower frequency and antenna size compels antenna researchers to compromise with antenna gain and efficiency for compliance with the CubeSat standards.

So, antenna design for nanosatellites has been a critical issue to the CubeSats researchers, especially for lower frequency [2]. Deployable wire antennas like monopoles dipoles, helical and Yagi–Uda arrays antennas are widely used in recent nanosatellite missions [3]. But mechanical deployment is quite sophisticated and this might increase the chance of mission failure [4]. Several nanosatellite missions have been failed due to antenna deployment complexity [5-7].

In contrast to the deployable antenna, patch antenna provides a low profile and improve the mission reliability; and makes the patch antennas are a good replacement of wire antennas. However, UHF patch antenna occupies large amount the nanosatellite body surface and introduce complexity to integrate sufficient solar cells. So, designing a small size ultra-high frequency (UHF) patch antenna strategically integrated with the satellite body and that do not require mechanical deployment has become a big challenge for nanosatellite and antenna researchers. Several research efforts have been devoted to designing a miniature UHF patch antenna with good impedance bandwidth, efficiency, and gain. Reactive impedance surface based U-slot patch antenna is one of them [8], where the antenna achieved lower UHF band (410–485 MHz) with antenna size reduction. However, the area of the antenna is 220× 220 × 20 mm^3 and the antenna is incompatible with CubeSat structure. In [9], to reduce the conventional RIS antenna size two-layer mushroom-like RIS is presented for 400 MHz UHF wireless communication, where the size is 66× 66 × 11.2 mm^3. Though the technique reduced the antenna size, antenna higher antenna height remains a critical issue for nanosatellite communication. Meander line technology facilitates achievement of the lower band with small antenna size [10]. However, this type of antenna does not work effectively when embedded in the complex structure and would degrade its radiation efficiency. To overcome this problem, epsilon-and-mu-near-zero (EMNZ) metamaterial has been developed and included in the ground plane of the antenna.

There have been a lot of research efforts since the last decades in the field of artificially engineered materials that show infrequent properties and do not readily exist in nature[11-14]. The unique properties metamaterial such as negative permittivity, permeability, refractive index or double negative characteristic have been utilized to improve antenna characteristics. Besides, metasurfaces structure, another form of engineered materials has been utilized to enhance antenna performance [15], linear to circular polarization convertion[16], and wavefronts control [17], etc. Now, researchers show their interest in another type of engineered material known as near-zero-metamaterials. This is the type of metamaterials whose metamaterial characteristics are near to zero like epsilon-near-zero (ENZ)),mu-near-zero (MNZ)), or both epsilon-and-mu-near-zero (EMNZ) [18,19]. Metamaterial with individual ENZ or MNZ shows impedances mismatch in the free space, which occurs high reflectance, high impedance and high loss [20]. However, EMNZ has low loss since the impedance is matched with free space. This type of metamaterial has been efficiently used in the field of antenna and wave propagation for enhancing the radiation efficiency, antenna size miniaturizing, coupling effect reduction, or for modifying the radiation patterns [20-23].

In this paper, an EMNZ metamaterial inspired printed patch antenna is proposed for the lower UHF communication system. This antenna is inspired from a conventional meander line patch antenna with EMNZ metamaterial ground plane to improve efficiency and impedance matching over the desired frequency range. Moreover, a technique using metamaterial array elements reduce the EM coupling with nanosatellite structure of the conventional meander line

antenna, while maintaining good impedance matching and efficiency for the UHF communication system. Moreover, the antenna is designed to fit into commercially available nanosatellite structures to mitigate antenna deployment complexity.

II. Antenna Design Methodology

The meander-line antenna concept is to fold the conductors back and forth to reduce antenna physical size. The resonant frequency of the conventional meander line antenna can be achieved by increasing the width of the meander line which leads the capacitance to the ground plane or increasing the meander line section to generate effective self-inductance for lower frequency adjustment. In this proposed design, the main parameters have been optimized by tuning line width, number of folds, the distance between folds to reduce the antenna size. As the physical dimension of the antenna is reduced, the antenna radiation efficiency and bandwidth are also decreased. To enhance radiation performance, EMNZ metamaterial structure has been used in the ground plane of the antenna. The performance of the proposed antenna has been characterized using CST microwave studio software and the prototype meander line antenna has been fabricated on Rogers 5800 dielectric, which has a thickness of 1.575 mm, a relative permittivity of 2.2, and loss tangent of 0.0013. The antenna geometry is depicted in Figure 1. The initial antenna considered partial ground plane-based meander line to achieve lower UHF frequency resonator. However, the partial ground plane has a direct effect on complex circuit and structure. The resonant frequency might shift and also reduce the efficiency while the antenna placed in a close proximity to the metallic structure [24,25]. To reduce this effect EMNZ metamaterial structure has been placed in the ground plane of the antenna. The array configuration of the metamaterial unit cell has been optimized until the desired antenna reflection coefficient and efficiency has been achieved. Moreover, the antenna has an omnidirectional radiation pattern, which has also been accomplished by the defected ground structure of EMNZ metamaterial array. The evolution stages are illustrated in Figure 2, and the corresponding reflection coefficient is presented in Figure 3. Figure 3 demonstrates that the operating frequency of the antenna has been tuned at 401 MHz and observed the corresponding reflection coefficient. The proposed antenna has been optimized for 401 MHz operating frequency with 80% total radiation efficiency and the optimized design parameters are listed in Table I.

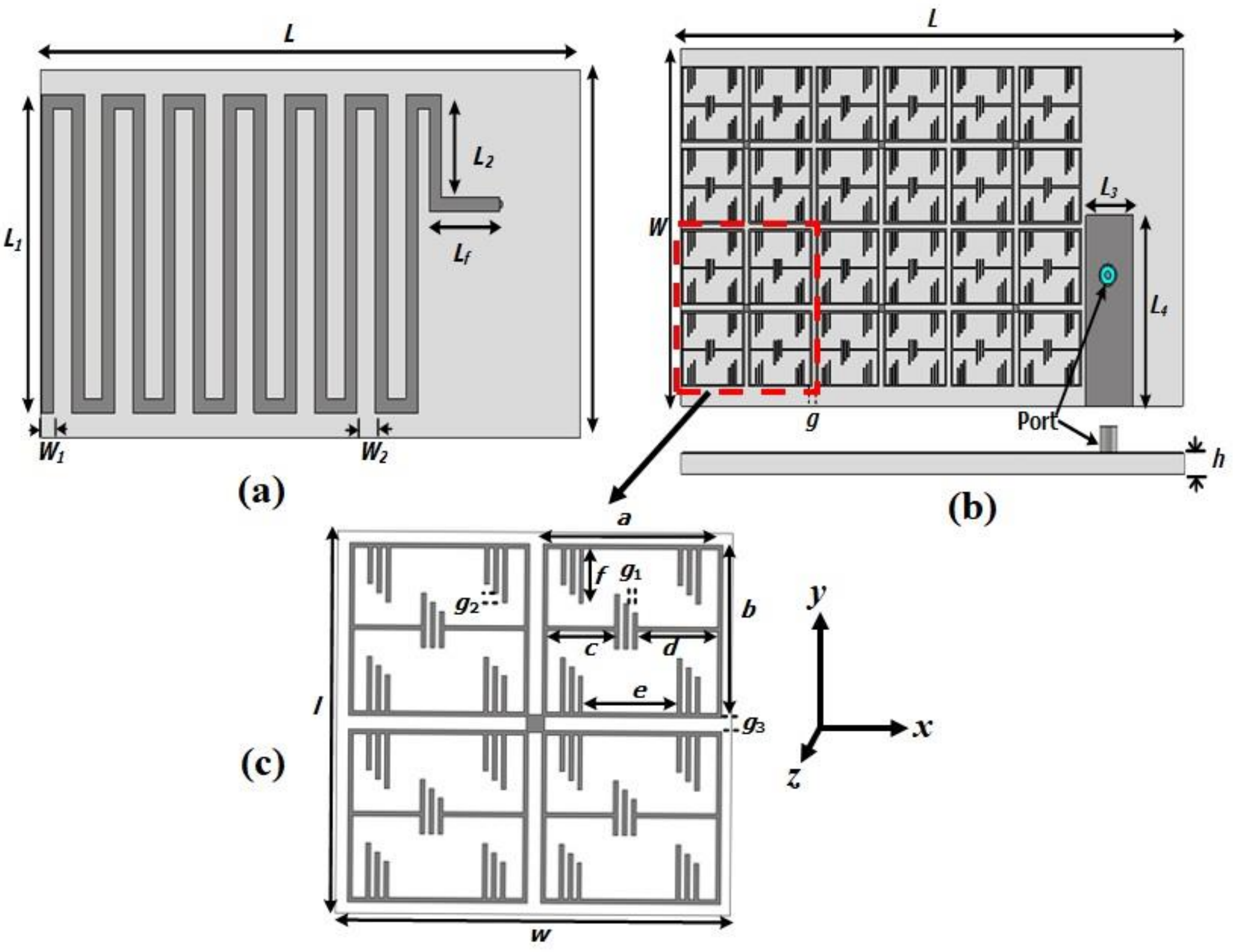

Fig. 1: Proposed metamaterial antenna configuration (a) Top view, (b) bottom view and (c) metamaterial structure

Fig. 2: Design evolution of the proposed UHF antenna: (a) antenna 1, (b) antenna 2, (c) antenna 3 and (d) antenna 4

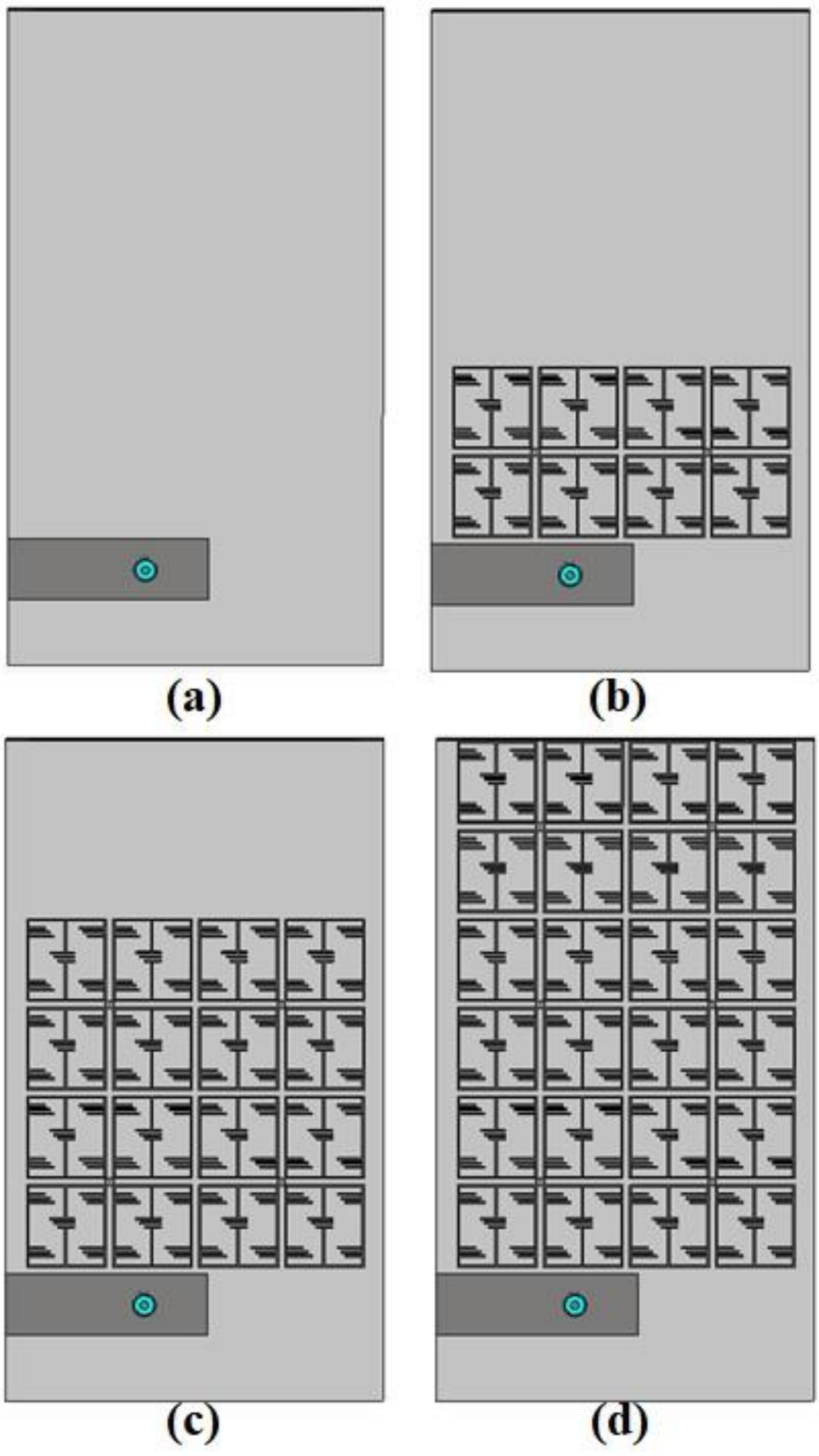

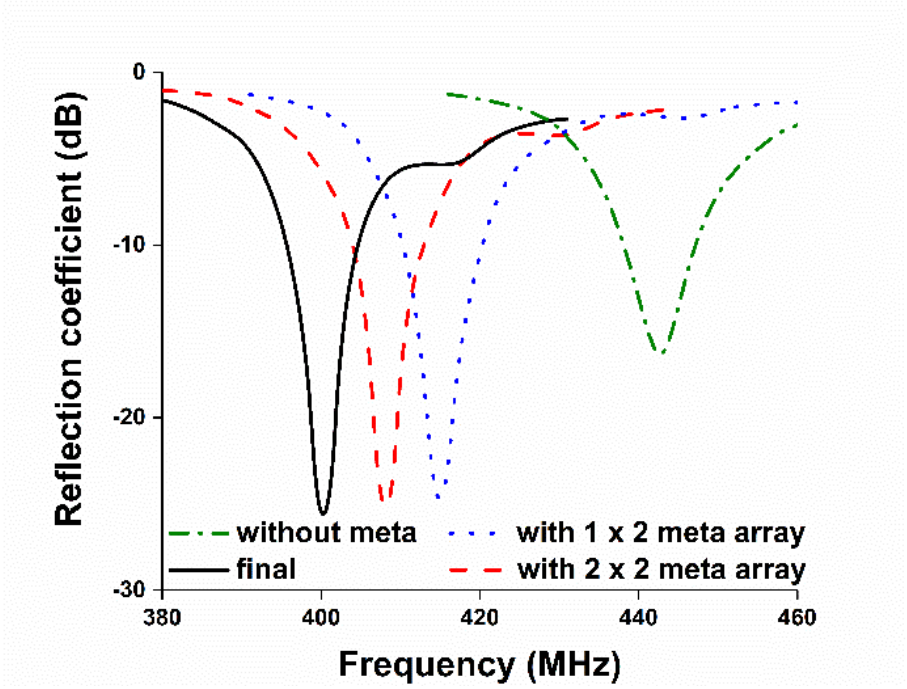

Fig. 3: Investigation on reflection coefficient at the different design stages

Table I: Optimized antenna design parameter

Parameter	Value (mm)	Parameter	Value (mm)
L	80	l	22.00
L_1	39.12	a	10.00
L_2	8.6	b	9.50
L_3	7.5	c	4.50
L_4	24	d	3.75
L_f	10.4	e	5.25
W	45	f	3.00
W_1	1.78	g_1	0.25
W_2	2.72	g_2	0.50
h	1.575	g_3	0.75
g	0.75		

III. EMNZ METAMATERIAL CHARACTERIZATION

The proposed metamaterial is composed of thin metallic arms and split resonant rings (SRRs). The outer metallic arms are utilized to realize electric resonance and effective ε-near-zero (ENZ), and the interconnected split ring resonators are used to realize magnetic resonance and effective μ-near-zero (MNZ), so the designed structure shows the properties of an impedance-matched near-zero-index metamaterial with ENZ and MNZ simultaneously. The metamaterial structure has been designed and simulated using CST Microwave Studio. Perfect electric conductor (PEC) and perfect magnetic conductor (PMC) boundary conditions are used along X and Y axis, respectively and two waveguide ports have been assigned in Z-direction according to [26,27]. The electromagnetic parameters of the proposed metamaterial have been retrieved using the following equation of robust metamaterial parameter extraction method [27,28]. In Figure 4, the metamaterial parameters for the unit cell are depicted, where it can be observed that the proposed structure shows permittivity, permeability and refractive index value of -0.005, 0.009, and -0.007, respectively. The metamaterials constitutive parameters show near-zero permittivity (ENZ), near-zero permeability (MNZ), and near-zero refractive index. Moreover, the metamaterial performance has been investigated for unit cell, 1×2, 2×2, and 3×2 array configuration using unit cell simulation setup, where 1×2-unit cell array, 2×2-unit cell array and 3×2- unit cell array configuration shows EMNZ metamaterial characteristics, depicted in Figure 5, Figure 6 and Figure 7, respectively. The summary of the study is listed in Table II and Table III.

$$S_{11} = \frac{R_{01}(1 - e^{i2nk_0d})}{1 - R^2{}_{01}e^{i2nk_0d}} \tag{1}$$

$$S_{21} = \frac{(1 - R^2{}_{01})e^{ink_0d}}{1 - R^2{}_{01}e^{i2nk_0d}} \tag{2}$$

$$R_{01} = \frac{z - 1}{z + 1} \tag{3}$$

$$z = \pm\sqrt{\frac{(1 + S_{11})^2 + S^2{}_{21}}{(1 - S_{11})^2 + S^2{}_{21}}} \tag{4}$$

$$X = \frac{1}{2S_{21}(1 - S^2{}_{11} + S^2{}_{21})} \tag{5}$$

$$e^{ink_0d} = X \pm i\sqrt{1 - X^2} \tag{6}$$

$$\eta = \frac{1}{k_0d}[\{[ln(e^{ink_0d})]'' + 2m\pi\} - i[ln(e^{ink_0d})]'] \tag{7}$$

$$\varepsilon = \frac{\eta}{z} \tag{8}$$

$$\mu = \eta z \tag{9}$$

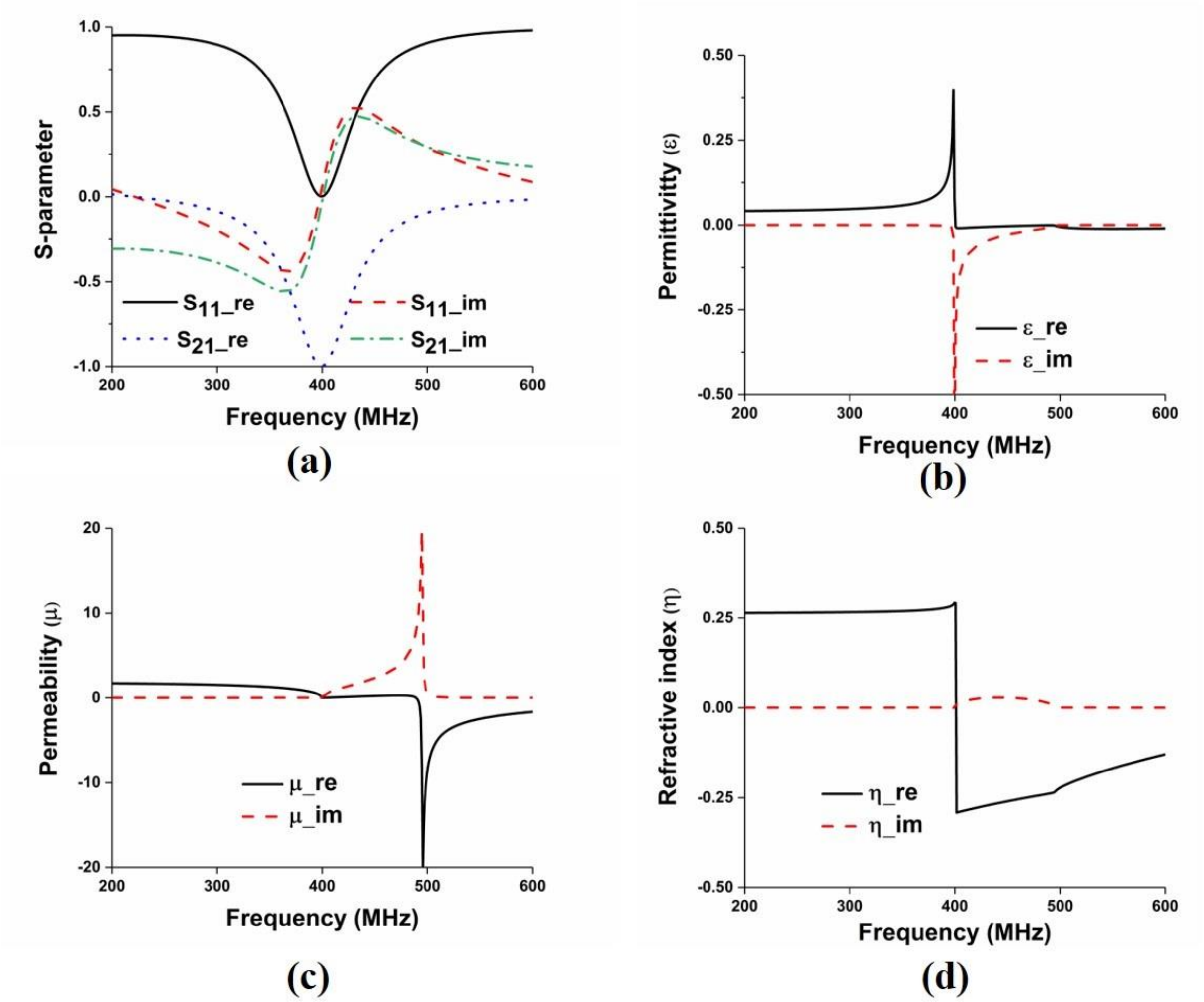

Fig. 4: (a). Metamaterial reflection and transmission coefficient for single unit cell; and retrieved metamaterial characteristics- (b). permittivity, (c). permeability and (d). refractive index.

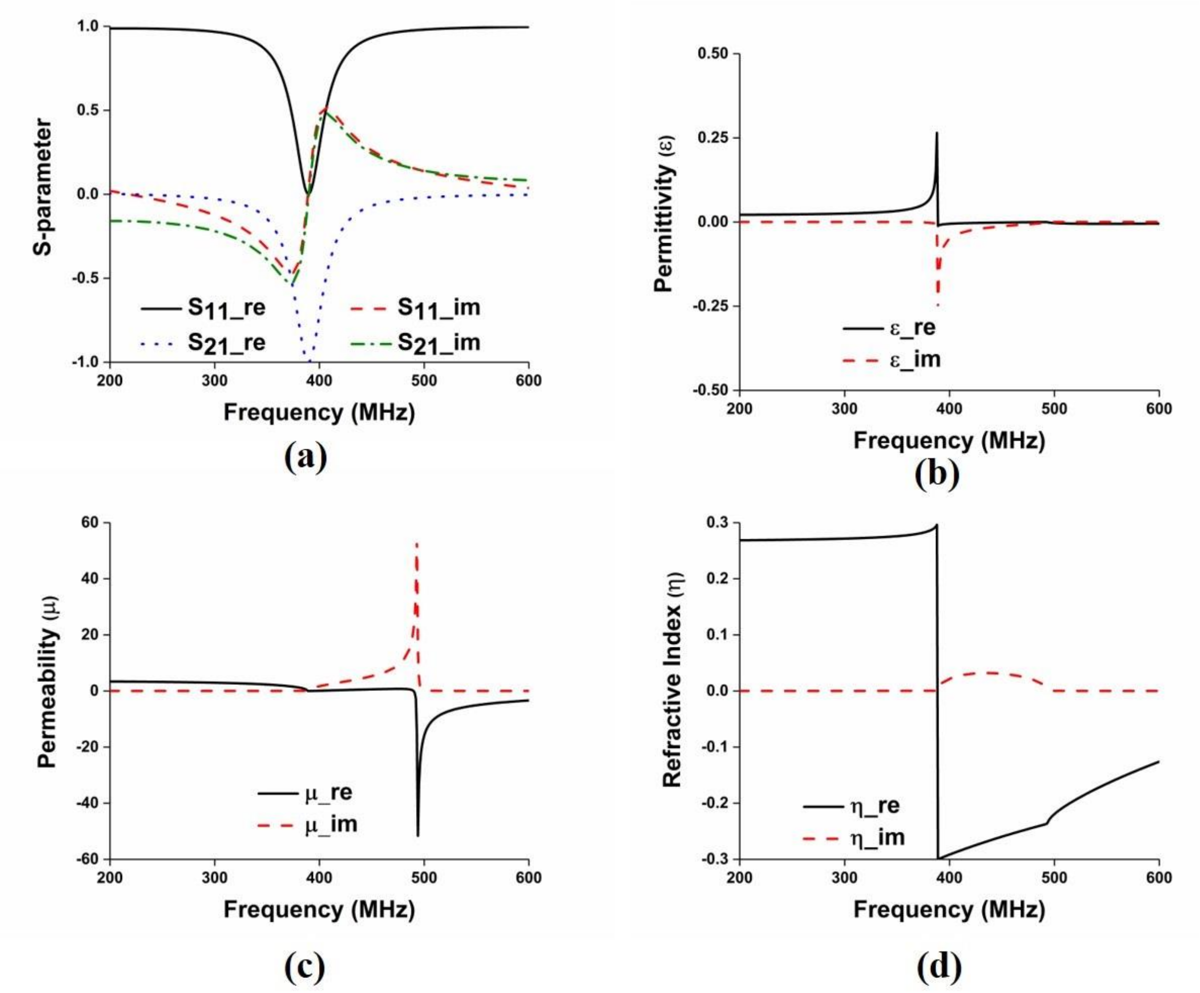

Fig. 5: (a). Metamaterial reflection and transmission coefficient for 1×2-unit cell array; and retrieved metamaterial characteristics- (b) permittivity, (c). permeability and (d). refractive index for 1×2-unit cell array.

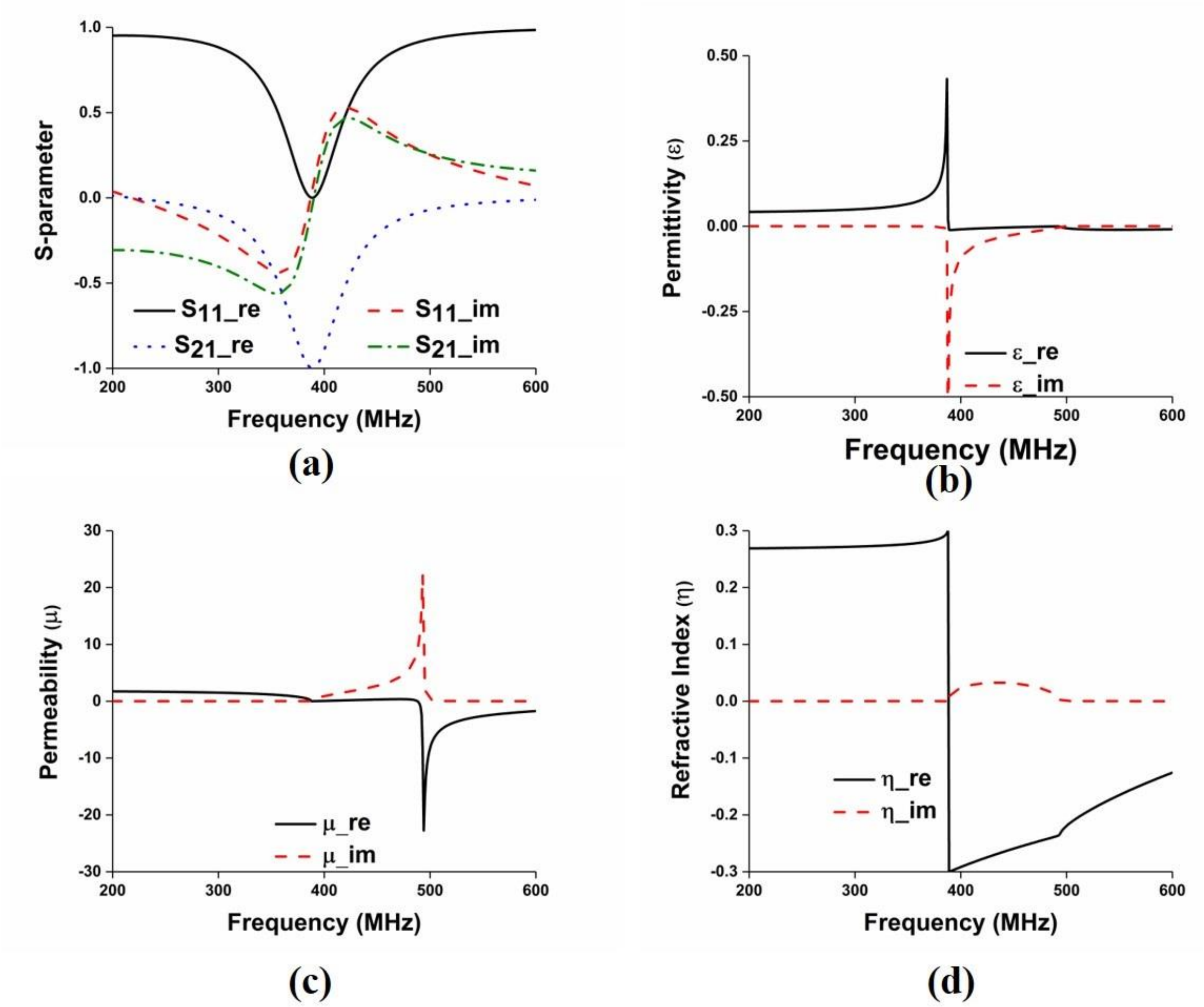

Fig. 6: (a). metamaterial reflection and transmission coefficient for 2×2-unit cell array; and retrieved metamaterial characteristics- (b) permittivity, (c). permeability and (d). refractive index for 2×2-unit cell array.

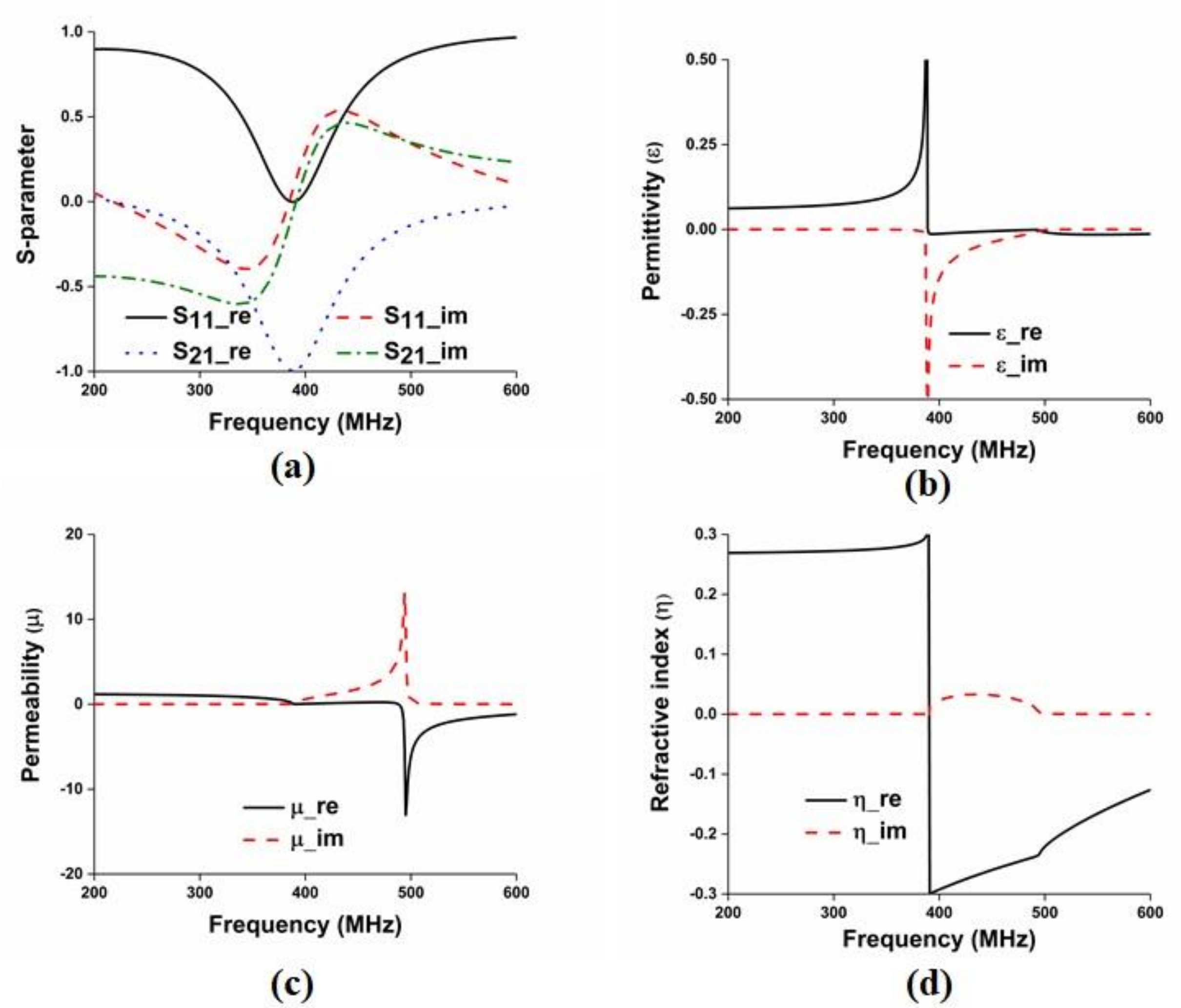

Fig. 7: (a). metamaterial reflection and transmission coefficient for 3×2-unit cell array; and retrieved metamaterial characteristics- (b) permittivity, (c). permeability and (d). refractive index for 3×2-unit cell array

Table II: EMNZ frequency region of the retrieved effective parameters.

Configuration	Parameter	EMNZ index frequency region (MHz)
	Permeability, μ	200-600
**Unit Cell**	Permittivity, ε	397-493
	Refractive index, *n*	200-600
	Permeability, μ	2000-600
**1×2-unit cell array**	Permittivity, ε	390-462
	Refractive index, *n*	200-600
	Permeability, μ	200-600
**2×2-unit cell array**	Permittivity, ε	389-490
	Refractive index, *n*	200-600
	Permeability, μ	200-600
**3×2-unit cell array**	Permittivity, ε	380-493
	Refractive index, *n*	200-600

Table III: EMNZ properties of the proposed metamaterial at 401 MHz

Configuration	Permittivity	Permeability	Refractive index
**Unit Cell**	-0.005	0.009	-0.007
**1×2-unit cell array**	-0.005	0.07	-0.29
**2×2-unit cell array**	-0.009	0.0542	-0.291
**3×2-unit cell array**	-0.013	0.039	-0.293

IV. ANTENNA PERFORMANCE ANALYSIS

The antenna has been fabricated according to the optimized parameters listed in Table I, illustrated in Figure 8. A 50 Ohm micro-miniature coaxial (MMCX) connector is connected to feed the antenna. The connector height is 4.5 mm from the ground plane and diameter is 2.54 mm, which is compatible with small ground plane and nanosatellite structure. The reflection coefficient of the proposed antenna has been measured using performance network analyzer (N5227A) that was calibrated using Electronic Calibration Module (N4694-60001). The simulated and measured reflection coefficient of the antenna has been analyzed, shown in Figure 9. The proposed antenna has achieved -10dB impedance bandwidth of 14.92 MHz (391 MHz – 405.92MHz).

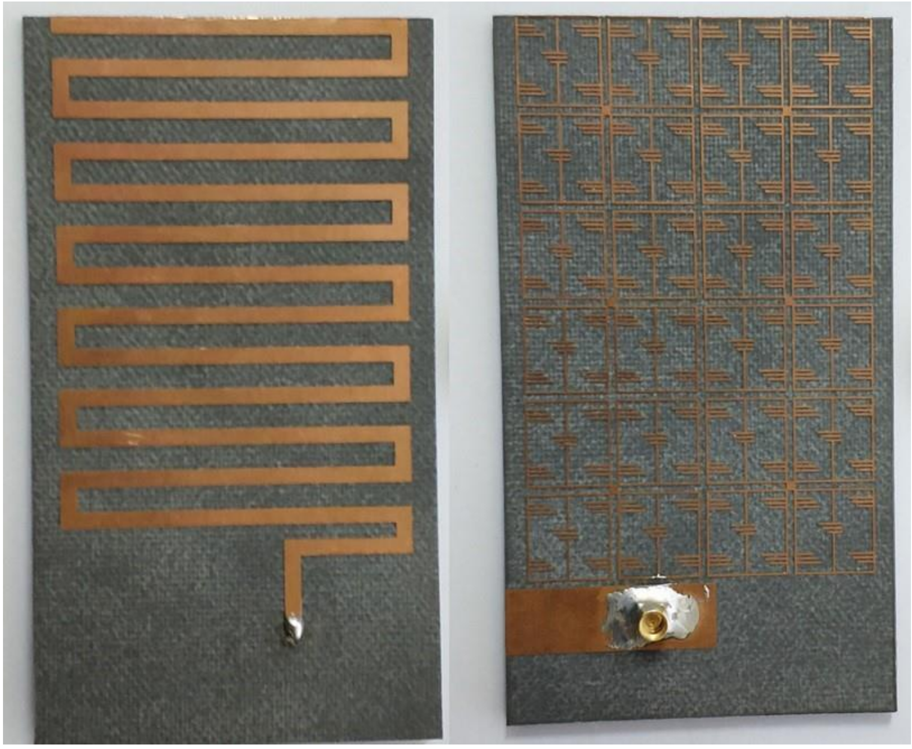

Fig. 8: Fabricated prototype of the optimized metamaterial antenna

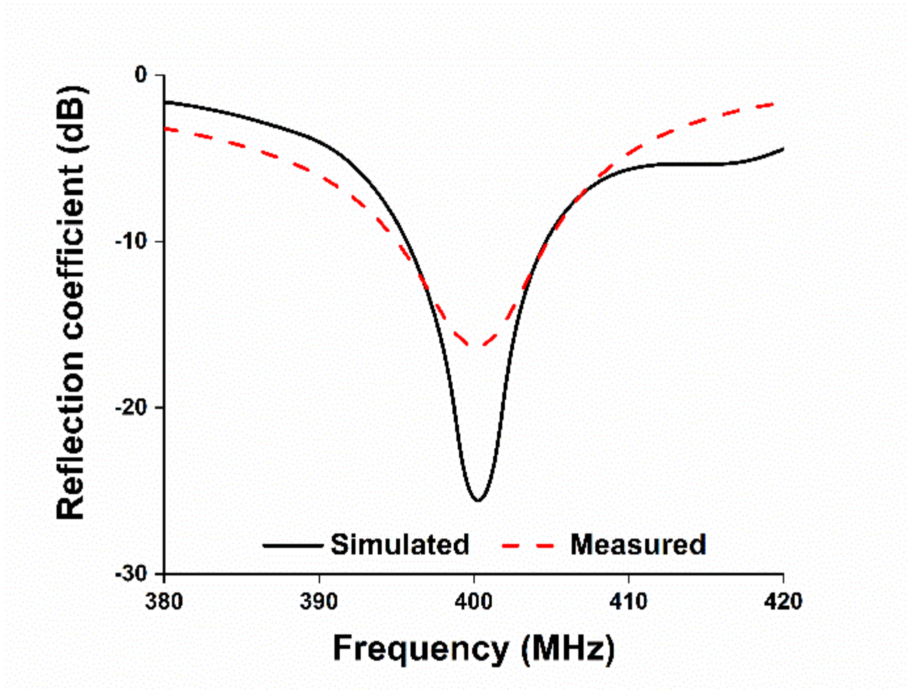

Fig. 9: Simulated and measured reflection coefficient

The surface current of the antenna without and with metamaterial has been investigated, shown in Figure 10. It is seen that a strong current is observed in meander line of the metamaterial antenna than without metamaterial antenna. It is predicted that the EMNZ structure has driven the surface current and act as strong radiation elements. Hence, the meander line with metamaterial radiated stronger radiation fields than without metamaterial antenna and contribute to improve the radiation efficiency and gain of antenna.

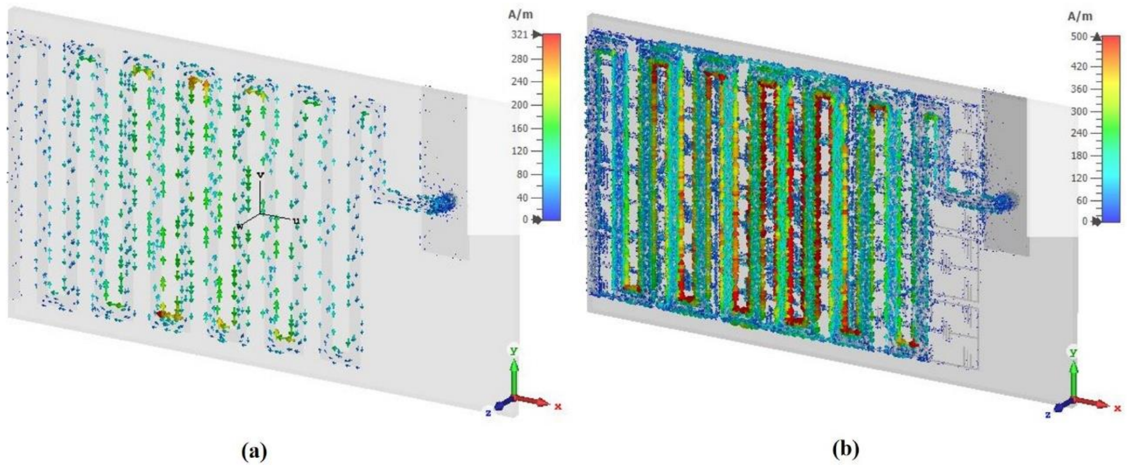

Fig. 10: Surface current distribution at 401MHz-(a) without metamaterial and (b) with metamaterial

The antenna far-field characteristics have been investigated using Satimo near-field measurement system. The simulated and measured radiation patterns at 401 MHz with and without metamaterial are presented in Figure 11. As it is expected, the antenna has approximately a dipole-like radiation pattern for conventional meander line antenna, Figure 11(a) and realized gain of 1.44 dB is observed at 401 MHz. Moreover, when the EMNZ array structure placed at the antenna relatively strong current distributions are seen on the meandered line. Thus, stronger radiation is observed in boresight direction and the radiation pattern shifted from conventional one. In addition, the antenna shows simulated realized gain of 1.77 dB and measured the gain of 1.65 dB. Both simulated and measured radiation patterns are in good agreement.

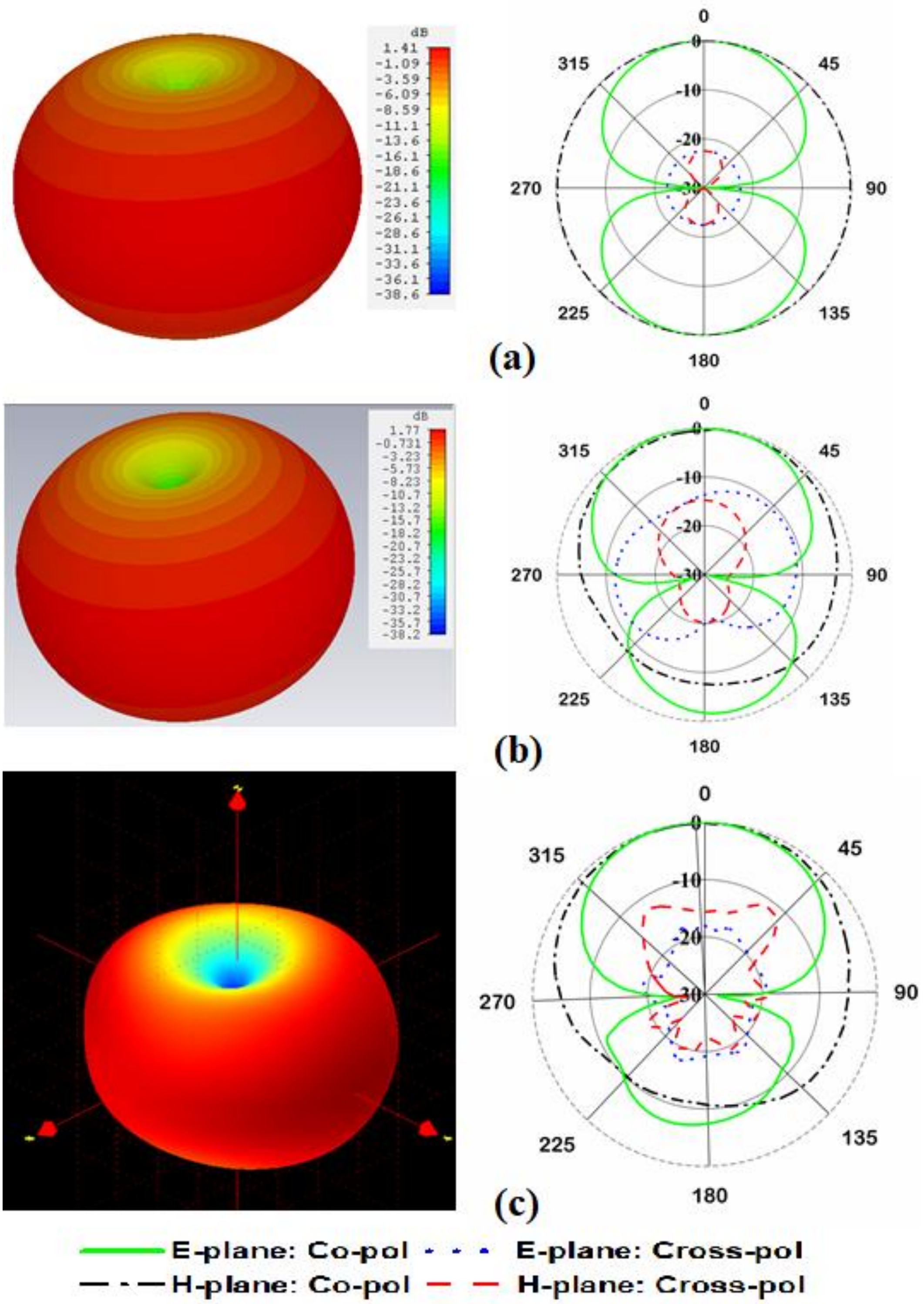

Fig. 11: Radiation pattern at 401 MHz- (a) simulated pattern without metamaterial. (b) simulated pattern with metamaterial and (c) measured pattern with metamaterial.

Antenna performance with Nanosatellite Structure

The antenna has been integrated with 2U nanosatellite structure, illustrated in Figure 12. The 2U nanosatellite structure was designed according to the Japan Aerospace Exploration Agency (JAXA) standard. The structure was considered as 227+/-0.1mm tall (+Z axis), 100+/- 0.1mm wide (+X, +Y axes) and maximum weight was 2.6 Kg. Aluminium 7075 material was considered for the nanosatellite frame structure. The solar panels are connected with the back-plane board of the structure, which is FR-4 substrate material. The antenna has been mounted on the backplane using RTV glue (Room-Temperature-Vulcanizing glue). The UHF antenna performance has been investigated with nanosatellite structure for both antenna 1 (without EMNZ) and antenna 4 (proposed). The antennas have been tuned at 401MHz with 2U nanosatellite structure, illustrated in Figure 13. The corresponding total efficiency of 45% and 57.7% has been realized for without EMNZ and with EMNZ structure, respectively. It is interesting to observe that the antenna total efficiency with 2U nanosatellite structure is improved by 12.7% after integrating EMNZ metamaterial structures.

In addition, the radiation pattern of the antenna with the nanosatellite structure has been investigated for both ‘with and without metamaterial’ antenna, shown in Figure 14. Both the radiation patterns are perturbed by nanosatellite structure and increased cross-polarization label with respect to free space condition. Compared with metamaterial-less (without metamaterial) antenna, the pattern is more spherical and provide more coverage of area of interest. Moreover, the realized gain obtained by without and with metamaterial antenna is 0.505 and 1.15 dB, respectively.

Moreover, the measured and simulated reflection coefficient bandwidth of the proposed antenna with 2U nanosatellite structure is 15 MHz (391-406 MHz) and 25 MHz (388-413.5 MHz), respectively. It is seen from Figure 15 that both results agree reasonably well, except little frequency shifting issue. Though both results ensure operating frequency, the resonant frequency discrepancy is acceptable considering fabrication and assembly tolerance. A summary of the antennas mounted on nanosatellite structure is tabulated in Table IV.

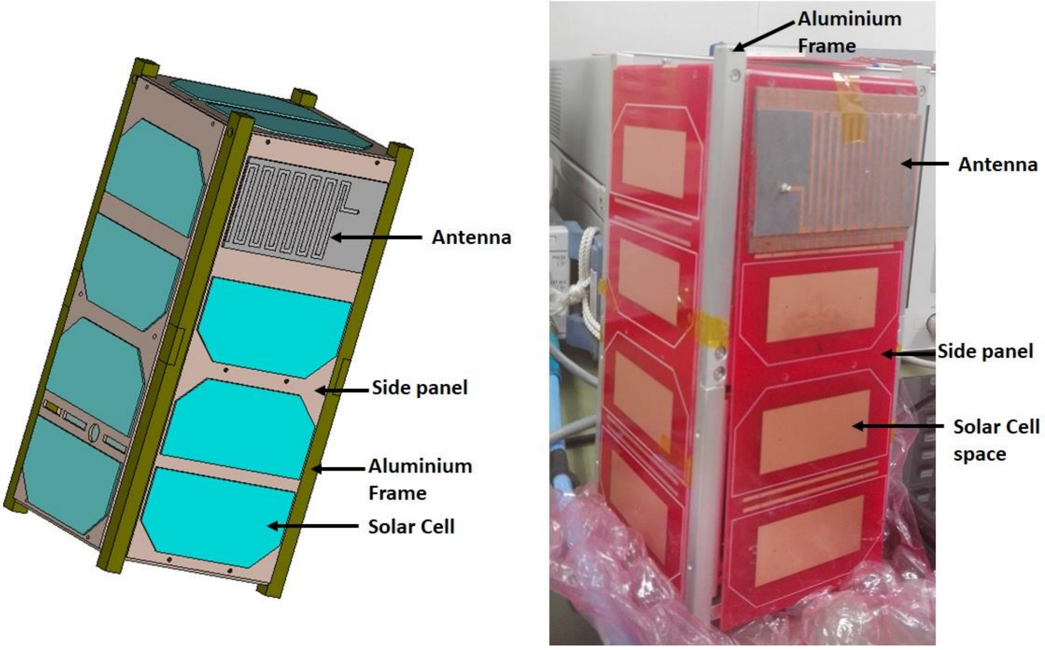

Fig. 12: Antenna attachment with 2U nanosatellite structure- (a) simulation and (b) fabricated.

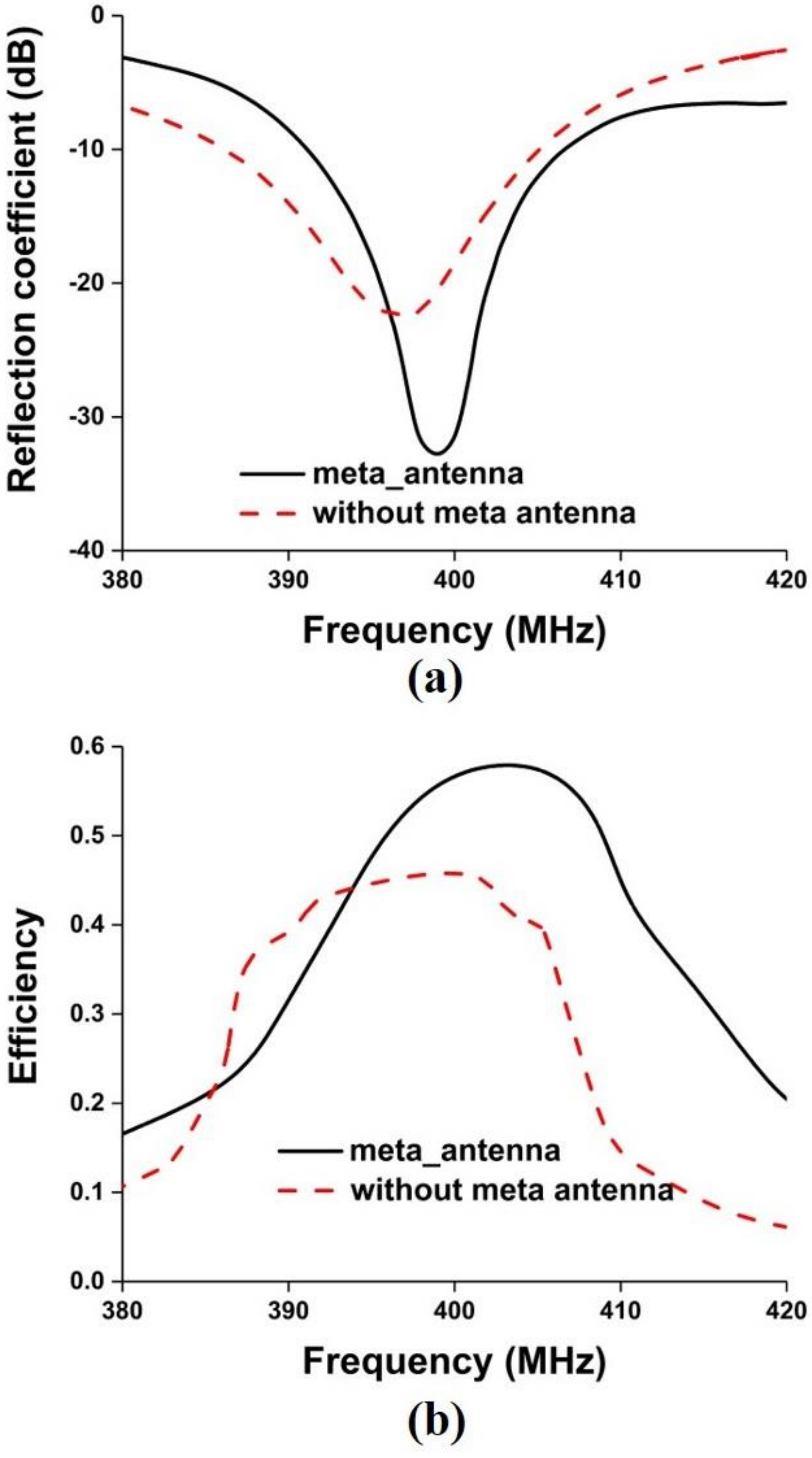

Fig. 13: Antenna performance with nanosatellite structure-(a) reflection coefficient and (b) total efficiency.

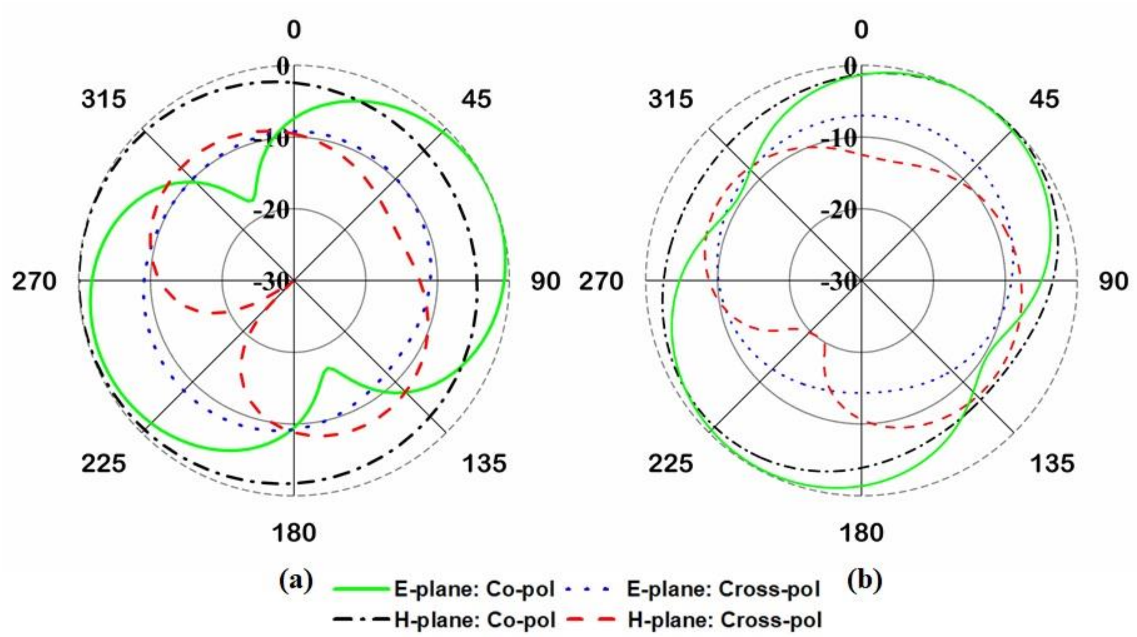

Fig. 14: Normalized radiation pattern of the proposed antenna with nanosatellite structure- (a) without metamaterial and (b) with metamaterial.

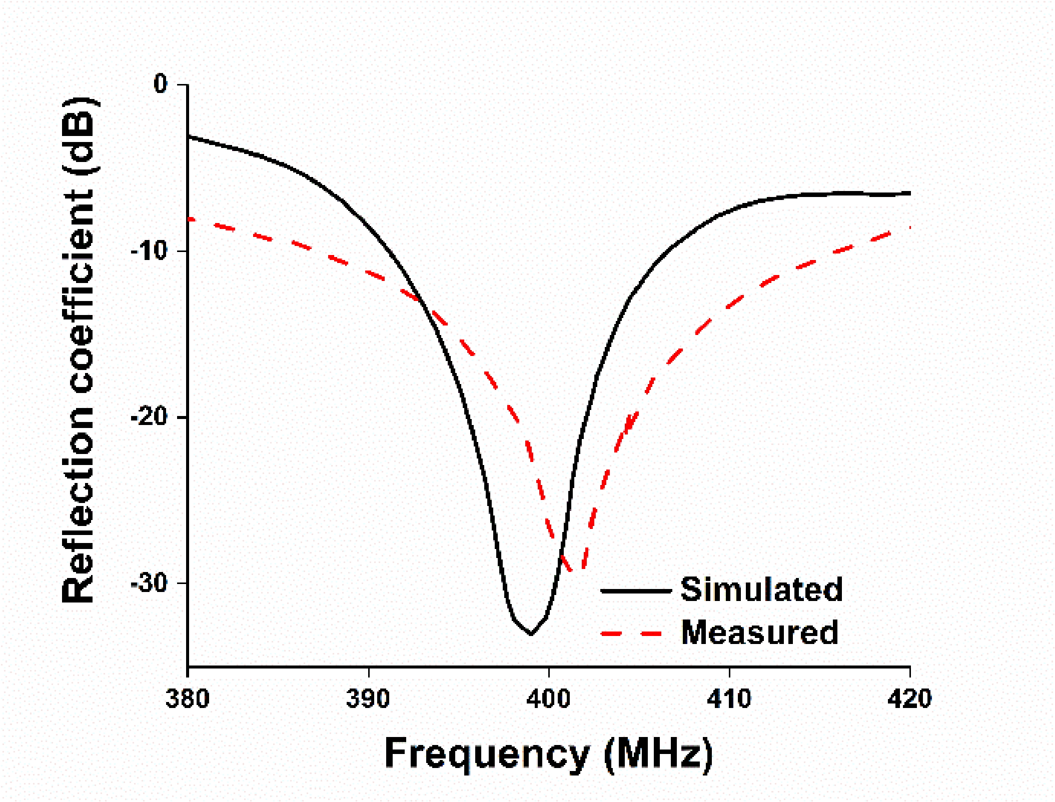

Fig. 15: Measured and simulated reflection coefficient of the proposed antenna with nanosatellite structure.

Table IV: A summary of the antennas mounted on nanosatellite structure

Antenna performance	Without metamaterial	With metamaterial
**Operating frequency (MHz)**	386-404.5	391 MHz – 405.92
**Realized gain (dB)**	0.505	1.15
**Efficiency (%)**	45	57.7
**Frequency shift with different structures (MHz)**	25-35	3-5

Antenna Signal Propagation Test

The Free Space Path Loss (FSPL) using variable attenuation has been conducted to estimate the maximum signal propagation of the proposed antenna with active nanosatellite. The FSPL has been calculated considering LEO orbit nanosatellite (400 km). The free space path loss has been calculated using the Friis transmission equation [29] and represented in equation (10).

$$FSPL = 20\ log_{10}\ (d) + 20\ log_{10}\ (f) + 20\ log_{10}\ \left(\frac{4\pi}{c}\right) - G_{Tx} - G_{Rx} \qquad (10)$$

Where *d* is the distance between receiver and transmitter end, *f* is the operating frequency, G_{Tx} is the transmitter antenna gain and G_{Rx} is receiver antenna gain.

The FSPL analysis using the proposed antenna is illustrated in Figure 16 (a). The signal modulation system is shown in Figure 16 (b). The active satellite with communication board has been placed on the anechoic chamber's turntable and proposed antenna (Tx) is transmitting signal. The receiving antenna (Rx) is positioned in the Horizontal orientation and connected to the Receiver through a variable attenuator. Attenuation increased gradually until the demodulation of the transmitted signal is no longer possible. The maximum attenuation value at which the receiver can demodulate the signal represents the signal strength of the transmitter antenna, which can address the maximum path loss. The SS power has been calculated by integrating over 100 kHz bandwidth. Care was taken to ensure that the RF power at receiver input never exceeds over -30dBm and RF power received by the demodulator doesn't exceed over -50 dBm for extended time period to protect the devices.

(a)

(b)

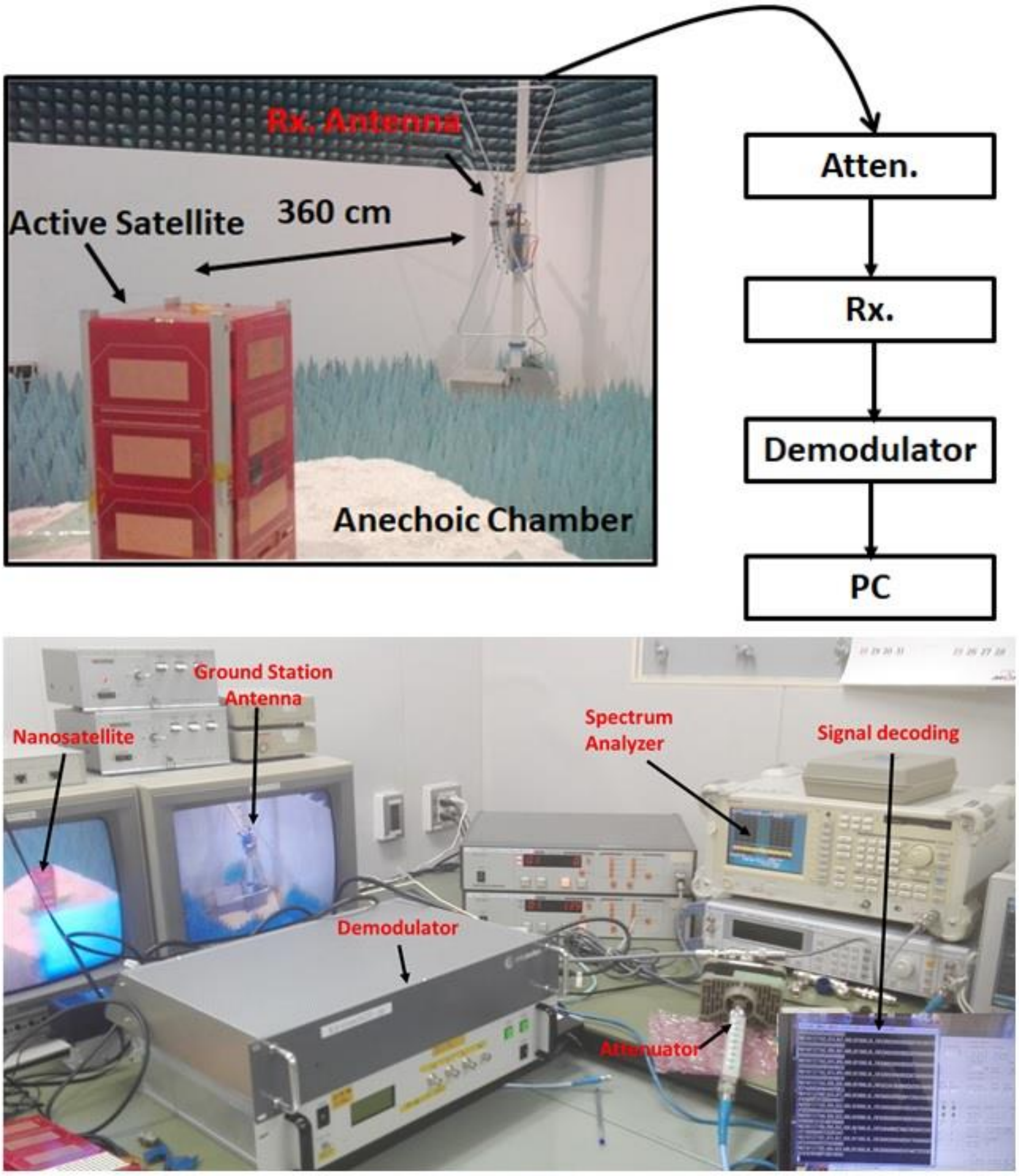

Fig. 16: FSPL performance investigation of the nanosatellite using proposed antenna, (a) measurement layout and (b) Signal demodulation

Gain of the proposed antenna at 401 MHz (G_{Tx}) =1.65 dBi

Gain of receiving antenna in anechoic chamber at 401 MHz (G_{Rx}) = 6.9 dBi

Ground station antenna gain at 401 MHz (G'_{Rx}) = 18 dBi

Free Space Path loss (FSPL) in the chamber (360 cm) at 401 MHz = 27.1 dB.

FSPL at orbital altitude (400 km) at 401 MHz = 116.8 dB

Extra attenuation required to achieve signal level estimation

= Orbital FSPL – FSPL(Chamber) - G'_{Rx} + G_{Rx}

= 116.8-27.1-18+6.9

= 78.6 dB

The attenuation level of the proposed antenna has been compared with conventional wire monopole antenna. From Figure 17, it is shown that the proposed antenna signal transmission capability is higher than wire monopole antenna. The receiver can demodulate the transmitting signal up to maximum attenuation of 98 dB.

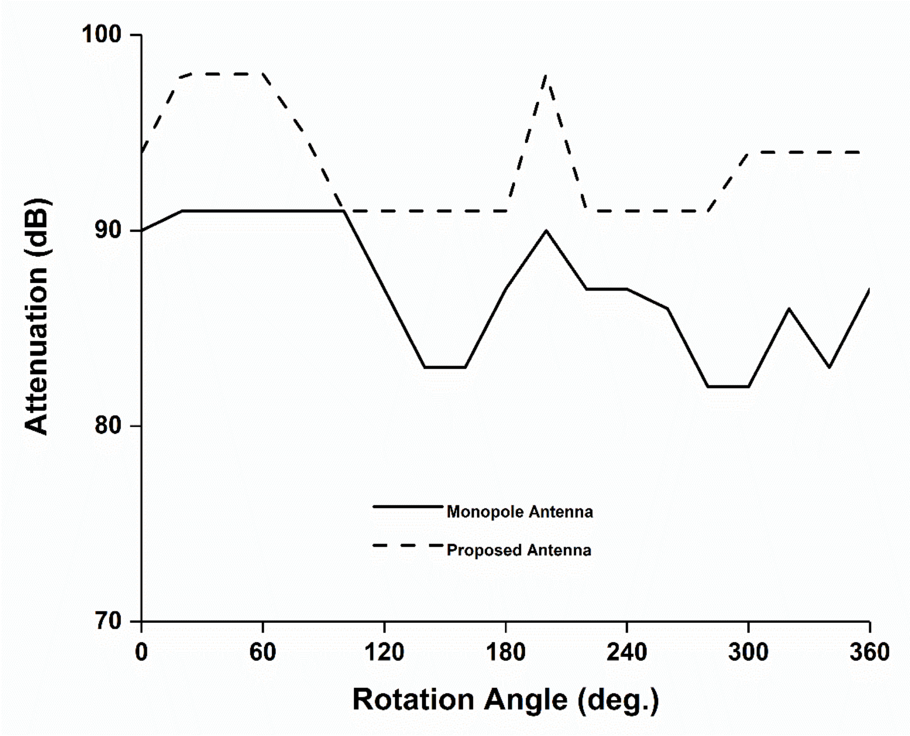

Fig. 17: Maximum FSPL analysis using the proposed antenna and wire monopole antenna

V. Discussion

The contributions of the proposed antenna are highlighted in this section. A comparison of the presented antenna with some existing UHF nanosatellite antennas is presented in Table V.

Deployment complexity free

RF failures in nanosatellite antenna have been reported by several incidents that have been occurred due to unsuccessful antenna deployment[6,30]. The established deployment mechanism is burn-wire method, but it is quite sophisticated to test in laboratory environment. The proposed patch antenna with compact size of 45×80 × 1.575 mm^3 can mitigate this issue.

Enhanced patch Antenna performance

The proposed antenna holds the potential for smooth communication between nanosatellite and the Earth, as it provides substantially higher gain and efficiency with only 45×80 mm^2 antenna dimension. The antenna addresses the frequency shifting issue that happens frequently in nanosatellite patch antenna and makes the mission risky[31].

Ensure smooth communication

This research also emphasised on the nanosatellite communication test using the antenna that proves the validity of the antenna's real-time performance. The satellite ground station receiver can demodulate the transmitting signal up to maximum attenuation of 98 dB, which is 20dB higher than required marginal Free Space Path Loss (FSPL). Moreover, the lab prototype of

the antenna has undergone a vibration test while embedded with nanosatellite structure that proves the robustness of the antenna.

Table V: A comparison of the presented antenna with different UHF nanosatellite antennas

**Reference**	**Antenna type & Size (mm)**	**Operating band (MHz)**	**Realized gain (dB)**	**Remarks**
Liu et al. [32]	Printed patch 320×80×3.17	427.38-437.17	2.12	incompatible with 1U and 2U structure due to larger antenna size
Kakoyiannis et al. [33]	Microstrip patch 170×120×6.4	435-437	0.7	incompatible with 1U and 2U structure due to larger antenna size
Podilchak et al. [34]	Microstrip Patch150×150×37	384-410	0.4	incompatible with 1U and 2U structure due to larger antenna size
Costantine et al. [35]	Deployable helix Helix heigh:500	365	8	High performance antenna but not compatible with 1U structure
Alam et al.[36]	3D-type antenna	398 MHz – 405 MHz	1.18	compatible with 1U and 2U structure but have design complexity
BIRDS-I CubeSat antenna [31,37,38]	Printed patch 72×32×1.575	418-448	0.55	compatible with 1U and 2U structure but mission incomplete due to low efficiency and frequency shifting issue
Proposed antenna	Printed patch 80×45×1.575	391 MHz – 405.92	1.77	compatible with 1U and 2U structure<br>Free from deployable complexity

VI. Conclusion

In this paper, an EMNZ metamaterial has been developed and integrated on the conventional meander line antenna ground plane to reduce the coupling effect with external auxiliary elements. The computational analysis of EMNZ metamaterial properties has been investigated for both unit cell and array configurations. The studies indicate that, compared with the conventional antenna, the proposed EMNZ structure has extended the antenna total efficiency by 12% without shifting resonant frequency. The free space path loss analysis has been performed to verify the feasibility of the proposed antenna for nanosatellite communication system.

Reference

1 Heidt, H., Puig-Suari, J., Moore, A., Nakasuka, S. and Twiggs, R., CubeSat: A new generation of picosatellite for education and industry low-cost space experimentation., *Logan, USA, 2000.*

2 Gao, S. *et al.* Antennas for modern small satellites. *IEEE Antennas and Propagation Magazine* **51** (2009).

3 Rahmat-Samii, Y., Manohar, V. & Kovitz, J. M. For Satellites, Think Small, Dream Big: A review of recent antenna developments for CubeSats. *IEEE Antennas and Propagation Magazine* **59**, 22-30 (2017).

4 Ernest, A. J., Tawk, Y., Costantine, J. & Christodoulou, C. G. A bottom fed deployable conical log spiral antenna design for CubeSat. *IEEE Transactions on Antennas and Propagation* **63**, 41-47 (2015).

5 Atcitty, S. AIHEC/TCU Advanced Manufacturing Network Initiative. (Sandia National Lab.(SNL-NM), Albuquerque, NM (United States), (2016).

6 Venturini, C., Braun, B., Hinkley, D. & Berg, G. Improving mission success of CubeSats. *Aerospace Corp., El Segundo, CA, USA, Aerospace Report No. TOR-2017-01689* (2017).

7 Fernandes, G., Santos, M., Silva, V., Almeida, J. and Nogueira, P., Thermal tests for cubesat in Brazil: Lessons learned and the challenges for the future. In Proceedings of the 67th International Astronautical Congress, Guadalajara (Vol. 27), Mexico, September 2016.

8 Sarabandi, K., Buerkle, A. M. & Mosallaei, H. Compact wideband UHF patch antenna on a reactive impedance substrate. *IEEE antennas and wireless propagation letters* **5**, 503 (2006).

9 Wu, J. & Sarabandi, K. Reactive impedance surface TM mode slow wave for patch antenna miniaturization [AMTA corner]. *IEEE Antennas and Propagation Magazine* **56**, 279-293 (2014).

10 Rashed, J. & Tai, C.-T. A new class of resonant antennas. *IEEE Transactions on Antennas and Propagation* **39**, 1428-1430 (1991).

11 Landy, N. I., Sajuyigbe, S., Mock, J., Smith, D. & Padilla, W. Perfect metamaterial absorber. *Physical review letters* **100**, 207402 (2008).

12 Enoch, S., Tayeb, G., Sabouroux, P., Guérin, N. & Vincent, P. A metamaterial for directive emission. *Physical Review Letters* **89**, 213902 (2002).

13 Zhang, K. *et al.* Phase-engineered metalenses to generate converging and non-diffractive vortex beam carrying orbital angular momentum in microwave region. *Optics express* **26**, 1351-1360 (2018).
14 Zhang, K., Ding, X., Wo, D., Meng, F. & Wu, Q. Experimental validation of ultra-thin metalenses for N-beam emissions based on transformation optics. *Applied Physics Letters* **108**, 053508 (2016).
15 Ullah, M. H. & Islam, M. T. A new metasurface reflective structure for simultaneous enhancement of antenna bandwidth and gain. *Smart materials and Structures* **23**, 085015 (2014).
16 Ratni, B., de Lustrac, A., Piau, G.-P. & Burokur, S. N. Electronic control of linear-to-circular polarization conversion using a reconfigurable metasurface. *Applied Physics Letters* **111**, 214101 (2017).
17 Ratni, B., de Lustrac, A., Piau, G.-P. & Burokur, S. N. Reconfigurable meta-mirror for wavefronts control: applications to microwave antennas. *Optics express* **26**, 2613-2624 (2018).
18 Mahmoud, A. M. & Engheta, N. Wave–matter interactions in epsilon-and-mu-near-zero structures. *Nature communications* **5**, 5638 (2014).
19 Dehbashi, R., Bialkowski, K. S. & Abbosh, A. M. Size Reduction of Electromagnetic Devices Using Double Near-Zero Materials. *IEEE Transactions on Antennas and Propagation* **65**, 7102-7109 (2017).
20 Liu, Y., Zhou, X., Zhu, Z. & Zhao, X. Broadband impedance-matched near-zero-index metamaterials for a wide scanning phased array antenna design. *Journal of Physics D: Applied Physics* **49**, 075107 (2016).
21 Soric, J. C., Engheta, N., Maci, S. & Alu, A. Omnidirectional Metamaterial Antennas Based on $\varepsilon $-Near-Zero Channel Matching. *IEEE Transactions on Antennas and Propagation* **61**, 33-44 (2013).
22 Yang, J. J., Francescato, Y., Maier, S. A., Mao, F. & Huang, M. Mu and epsilon near zero metamaterials for perfect coherence and new antenna designs. *Optics express* **22**, 9107-9114 (2014).
23 Ullah, M. H. *et al.* ZIM cover for improvement of the bandwidth and gain of patch antenna. *Current Applied Physics* **16**, 837-842 (2016).
24 Qing, X. & Chen, Z. N. Proximity effects of metallic environments on high frequency RFID reader antenna: Study and applications. *IEEE transactions on Antennas and Propagation* **55**, 3105-3111 (2007).
25 Ukkonen, L., Sydanheimo, L. & Kivikoski, M. Effects of metallic plate size on the performance of microstrip patch-type tag antennas for passive RFID. *IEEE Antennas and Wireless Propagation Letters* **4**, 410-413 (2005).
26 Zarghooni, B., Dadgarpour, A. & Denidni, T. A. Greek-key pattern as a miniaturized multiband metamaterial unit-cell. *IEEE Antennas and Wirel. Propag. Lett* **14**, 1254-1257 (2015).
27 Islam, M. T., Bin Ashraf, F., Alam, T., Misran, N. & Singh, M. S. J. Investigation of Left-Handed Meta-Atom for Radio Frequency Shielding Application. *Science of Advanced Materials* **10**, 1582-1587 (2018).
28 Chen, X., Grzegorczyk, T. M., Wu, B.-I., Pacheco Jr, J. & Kong, J. A. Robust method to retrieve the constitutive effective parameters of metamaterials. *Physical review E* **70**, 016608 (2004).
29 Saakian, A. *Radio wave propagation fundamentals*. (Artech House, 2011).
30 Saunders, A. A Failure Analysis of the EXOCUBE CubeSat, *13th Annual Cubesat Workshop* ,San Luis Obispo, USA, 20-04-2016.

31 Samsuzzaman, M., Islam, M. T., Kibria, S. & Cho, M. BIRDS-1 CubeSat Constellation Using Compact UHF Patch Antenna. *IEEE Access* **6**, 54282-54294 (2018).
32 Liu, X. *et al.* Transparent and Nontransparent Microstrip Antennas on a CubeSat: Novel low-profile antennas for CubeSats improve mission reliability. *IEEE Antennas and Propagation Magazine* **59**, 59-68 (2017).
33 Kakoyiannis, C. G. & Constantinou, P. A compact microstrip antenna with tapered peripheral slits for CubeSat RF Payloads at 436MHz: Miniaturization techniques, design & numerical results, *IEEE International Workshop on Satellite and Space Communications(IWSSC)*, 255-259 (*2008*).
34 Podilchak, S. K., Murdoch, A. P. & Antar, Y. M. Compact, Microstrip-Based Folded-Shorted Patches: PCB antennas for use on microsatellites. *IEEE Antennas and Propagation Magazine* **59**, 88-95 (2017).
35 Costantine, J. *et al.* UHF deployable helical antennas for CubeSats. *IEEE Transactions on Antennas and Propagation* **64**, 3752-3759 (2016).
36 Alam, T. *et al.* Design and compatibility analysis of a solar panel integrated UHF antenna for nanosatellite space mission. *PloS one* **13**, e0205587 (2018).
37 Basyirah, S. BIRDS-2 Antenna Selection Design & Configuration. *BIRDS Project Newsletter*, 25-29 (2017).
38 *Nanosatellite & CubeSat Database | Missions, constellations*, <https://www.nanosats.eu/> (2019).

Acknowledgements
This research is a collaboration work between Universiti Kebangsaan Malaysia and the Laboratory of Spacecraft Environment Interaction Engineering (LaSEINE), Kyushu Institute of Technology, Japan. This work is supported by the research university grant of Universiti Kebangsaan Malaysia.

Author Contributions
The antenna design and analysis were performed by Touhidul Alam. Prototype fabrication, characterization experiment and antenna performance characterization were conducted by Touhidul Alam, and Mohammad Tariqul Islam. The experimental set-up for nanosatellite structure was established by Mengu Cho. The project was supervised by Mohammad Tariqul Islam.

Additional Information
Competing interests: The authors declare no competing financial and/or non-financial interests.

Figure legend:

Fig. 1: Proposed metamaterial antenna configuration (a) Top view, (b) bottom view and (c) metamaterial structure.

Fig. 2: Design evolution of the proposed UHF antenna.

Fig. 3: Investigation on reflection coefficient at the different design stages.

Fig. 5: (a). Metamaterial reflection and transmission coefficient for 1×2-unit cell array; and retrieved metamaterial characteristics- (b) permittivity, (c). permeability and (d). refractive index for 1×2-unit cell array.

Fig. 6: (a). metamaterial reflection and transmission coefficient for 2×2-unit cell array; and retrieved metamaterial characteristics- (b) permittivity, (c). permeability and (d). refractive index for 2×2-unit cell array

Fig. 7: (a). metamaterial reflection and transmission coefficient for 2×2-unit cell array; and retrieved metamaterial characteristics- (b) permittivity, (c). permeability and (d). refractive index for 3×2-unit cell array

Fig. 8: Fabricated prototype of the optimized metamaterial antenna.

Fig. 9: Simulated and measured reflection coefficient.

Fig. 10: Surface current distribution at 401MHz-(a) without metamaterial and (b) with metamaterial

Fig. 11: Radiation pattern at 401 MHz- (a) simulated pattern without metamaterial.

(b) simulated pattern with metamaterial and (c) measured pattern with metamaterial.

Fig. 12: Antenna attachment with 2U nanosatellite structure- (a) simulation and (b) fabricated

Fig. 13: Antenna performance with nanosatellite structure-(a) reflection coefficient and (b) total efficiency

Fig. 14: Normalized radiation pattern of the proposed antenna with nanosatellite structure-

(a) without metamaterial and (b) with metamaterial.

Fig. 15: Measured and simulated reflection coefficient of the proposed antenna with nanosatellite structure

Fig. 16: FSPL performance investigation of the nanosatellite using proposed antenna

Fig. 17: Maximum FSPL analysis using the proposed antenna and wire monopole antenna

Table legend:

Table I: Optimized antenna design parameter.

Table II: EMNZ frequency region of the retrieved effective parameters.

Table III: EMNZ properties of the proposed metamaterial at 401 MHz.

Table IV: A summary of the antennas mounted on nanosatellite structure

Table V: A comparison of the presented antenna with different UHF nanosatellite antennas
